# Supplementary material for: Disentangling associations between pubertal development, healthy activity behaviors, and sex in adolescent social networks
Source: PLoS One. 2024 May 16;19(5):e0300715. doi: 10.1371/journal.pone.0300715 (PMC11098364; doi:10.1371/journal.pone.0300715)
Supplement: S1 File — (DOCX) [file pone.0300715.s001.docx]

**Supporting Information**

Disentangling associations between pubertal timing, healthy activity behaviors, and sex in adolescent social networks.

Mark C. Pachucki, Lindsay Till Hoyt, Li Niu, Richard Carbonaro, Hsin Fei Tu, John R. Sirard, Genevieve Chandler

Part 1. Data preparation and sample model code…………………………………………………………… 1

Part 2. Goodness of Fit diagnostics for Sunshine High and Jefferson High models……………….………. 6

**Part 1. Data preparation and sample model code**

**1. Data preparation of covariate data (example school given)**

//In Stata, generate main merged covar file from desired covars, then keep only obs from specific school:

keep if SCID=="077" /*this leaves 1721 observations within school 077*/

save "school077_covarsw1.dta", replace

***** OUTCOMES *******

//(a) Physical activity - freq per week

//pawkw1

//

//(b) Screen Time - hrs per week

//sthrsw1

//

//(c) Sleep - original covars have limited # of values (<11)

//slphrsw1

//slpenoughw1

***** PREDICTORS *******

//r_pubertyw1 (relative puberty)

//pubertyw1raw (Stage-normative (raw) puberty/physical development)

***** COVARIATES *******

//Sociodemographics & other confounders

rename FR_FLAG fr_flag

// malew1 - already binary

tab gradew1

//we're going to drop these students

drop if gradew1==7 | gradew1==8 /*n=13, but also 7 in 9th grade who get dropped too*/

drop if gradew1==99 /*n=7*/

drop if gradew1==97 /*n=50*/

//racew1 - categorical, but need to inspect distributions and check for small cells

quietly tabulate racew1, generate(racew1_)

rename racew1_1 white

rename racew1_2 black

rename racew1_3 natam

rename racew1_4 asianpi

rename racew1_5 other

//alternate: combine natam & other into one category

gen racew1_alt=racew1

replace racew1_alt=5 if racew1_alt==3

quietly tabulate racew1_alt, generate(racew1_alt)

rename racew1_alt4 othernatam

label variable othernatam `"racew1_alt==other or native american"'

//peducw1 - 5 categories, ordinal

//zbmiowobw1 - 2 categories

//agew1 - continuous between 13-21

//sympcntw1 - continous between 0-65.5

// //quick check for any data abberations

// tab1 pawkw1 sthrsw1 slpenoughw1 slphrsw1

// tab1 r_pubertyw1 pubertyw1raw

// tab1 malew1 gradew1 white black natam asianpi other hispanicw1 peducw1 zbmiowobw1 agew1 fr_flag sympcntw1

//

// //quick check for likely collinearities

// tab agew1 gradew1

// tab malew1 pubertyw1raw

// tab whitehw1 pubertyw1raw

// tab agew1 zbmiowobw1

// tab pubertyw1raw zbmiowobw1

// quick check for missing covars

egen nmiss = rowmiss(pawkw1 sthrsw1 slpenoughw1 slphrsw1 ///

r_pubertyw1 pubertyw1raw ///

malew1 gradew1 peducw1 zbmiowobw1 agew1 fr_flag ///

racew1 whitehw1 blackhw1 asianhw1 othernat hisphw1)

tab nmiss

//assess which covars are missing the most data

mdesc pawkw1 sthrsw1 slpenoughw1 slphrsw1 ///

r_pubertyw1 pubertyw1raw ///

malew1 gradew1 peducw1 zbmiowobw1 agew1 fr_flag ///

racew1 whitehw1 blackhw1 asianhw1 othernat hisphw1

// Variable | Missing Total Percent Missing

// ----------------+-----------------------------------------------

// pawkw1 | 0 1,658 0.00

// sthrsw1 | 3 1,658 0.18

// slpenoughw1 | 3 1,658 0.18

// slphrsw1 | 3 1,658 0.18

// r_pubertyw1 | 19 1,658 1.15

// pubertyw1raw | 26 1,658 1.57

// malew1 | 0 1,658 0.00

// gradew1 | 0 1,658 0.00

// peducw1 | 56 1,658 3.38

// zbmiowobw1 | 0 1,658 0.00

// agew1 | 0 1,658 0.00

// fr_flag | 0 1,658 0.00

// racew1 | 9 1,658 0.54

// whitehw1 | 6 1,658 0.36

// blackhw1 | 6 1,658 0.36

// asianhw1 | 6 1,658 0.36

// othernatam | 9 1,658 0.54

// hisphw1 | 6 1,658 0.36

// ----------------+-----------------------------------------------

keep AID pawkw1 sthrsw1 /// pa / st

r_pubertyw1 sn_pubertyw1 pubertyw1raw menarchew1 /// puberty

slpenoughw1 slphrsw1 ///sleep

gradew1 zbmiowobw1 agew1 malew1 peducw1 ///age/sociodem

racew1 racew1_alt white black natam asianpi other othernat hispanicw1 fr_flag nmiss

order AID pawkw1 sthrsw1 slphrsw1 slpenoughw1 ///

r_pubertyw1 sn_pubertyw1 pubertyw1raw menarchew1 gradew1 agew1 malew1 ///

peducw1 racew1 racew1_alt white black natam asianpi other othernatam hispanicw1 fr_flag

save "/data_nb/Analytic datasets/ERGM/School077/school077_covars_w1.dta", replace

//this is the version with complete covariate data (no missings have been dropped)

//n=1658

drop if nmiss > 0

//n=1568 individuals remain with complete (no missing) covariate data

//complete cover data is necessary for ERGMs

save "/data_nb/Analytic datasets/ERGM/School077/school077_covars_w1_nm.dta", replace

**2. Data preparation of network ties**

A. In Stata

//Identify nominated friends from in-home survey data

use "hfriend1.dta", replace

//Rename in-home friend variables

forvalues i=1/5 {

rename mf_aid`i' w1_mf_aid`i'

rename ff_aid`i' w1_ff_aid`i'

}

//relabel in-home variables

forvalues i=1/5 {

label variable w1_mf_aid`i' "MALE FRIEND `i' - HOME DATA, W1"

label variable w1_ff_aid`i' "FEMALE FRIEND `i' - HOME DATA, W1"

}

//recode to be double type - already checked the string matched the new var

forvalues i=1/5 {

destring w1_mf_aid`i', replace

destring w1_ff_aid`i', replace

}

//Next, generate a simple dyad list across all in-home schools

//This generates a file with Ego, Alter, school Id, alter #

//need to merge school question data with dyadic data

merge 1:m AID using "schoolw1.dta", gen(mw1sch)

keep AID SCID SAT_SCHL graderw1 w1_mf_aid1-w1_ff_aid5 flgw1

keep if SAT_SCHL==1 & flgw1==1

drop SAT_SCHL

rename w1_ff_aid1 w1_mf_aid6 /*renumber these ties to create alters 1-10 */

rename w1_ff_aid2 w1_mf_aid7

rename w1_ff_aid3 w1_mf_aid8

rename w1_ff_aid4 w1_mf_aid9

rename w1_ff_aid5 w1_mf_aid10

sort SCID AID

reshape long w1_mf_aid, i(AID SCID) j(alt_n)

sort SCID AID alt_n

rename AID ego_w1

rename w1_mf_aid alter_w1

order ego* alter*

#subset to just school 077

keep if SCID=="077" /*retains n=11,670 observations*/

replace alter=. if alter==77777777 | alter==55555555 | alter==88888888 | alter==99999999

drop SCID alt_n

gen wght=.

replace wght=1 if alter!=. /* weight each tie evenly (1)*/

sort ego

destring ego, replace

tab graderw1 wave if wght==1 /*inspect tie distribution - looks like a few 8th/9th graders */

drop if (graderw1==8 | graderw1==9)

save “077_dyadlist_w1.dta”

B. In R, generate adjacency matrix

rm(list = ls())

library(foreign)

library(rio)

library(network)

library(readstata13)

library(ergm)

library(sna)

library(stargazer)

library(dplyr)

'%!in%' <- function(x,y)!('%in%'(x,y))

#import Wave 1 dyadlist generated in stata

data_77 <-read.dta13("077_dyadlist_w1.dta")

#read in covariate data

var_77 <- covars_077_w1 <-read.dta13("school077_covars_w1_nm.dta")

#this is n=1568 observations

# check the number of covariates in the network

node_list = unique(c(data_77$ego_w1, data_77$alter_w1))

aid_check = list()

for( i in var_77$AID){

if(i %!in% node_list){

aid_check = append(aid_check, i)

}

}

# check the number of network nodes in the covariates

dyad_check = list()

for(i in node_list){

if(i %!in% var_77$AID){

dyad_check = append(dyad_check, i)

}

}

# generate a list of IDs who are in both network and the covariates

g1 = list()

for(i in var_77$AID){

if (i %in% node_list){ g1 = append(g1, i)} else {next}

}

g1 = unlist(g1)

g1 = as.character(g1)

# generate a sub network dataset with selected nodes only

s1 = data_77[data_77$ego_w1 %in% g1,]

s2 = s1[s1$alter_w1 %in% g1,1:2]

s2$ego_w1 <- as.character(s2$ego_w1)

s2$alter_w1 <- as.character(s2$alter_w1)

n = length(g1)

# generate the adjacency matrix

adj_77 = matrix(0, n, n)

for(z in 1:dim(s2)[1]){

i = match(s2$ego_w1[z],g1)

print(i)

j = match(s2$alter_w1[z], g1)

adj_77[i,j] = 1

}

sum(adj_77)

sum(s1)

rownames(adj_77) <- g1

colnames(adj_77) <- g1

#symmetrize graph (weak), so that: i<->j iff i->j or i<-j (OR rule)

n77_symw <- as.network.matrix(adj_77, type = "adjacency",directed = F)

n77_symw %v% 'vertex.names' <- as.character(n77_symw %v% 'vertex.names')

var_77$AID <- as.character(var_77$AID)

#read in modular VIF function to test for multicollinearity (see Duxbury 2021 appendix)

source("/Duxbury_VIF_ERGM_modular.R")

**3. Assignment of covariate data to network object for ERGM**

# assign attributes for the network

n77_symw %v% 'pawkw1' <- var_77$pawkw1_v2[match(n77_symw %v% 'vertex.names',var_77$AID)]

n77_symw %v% 'sthrsw1' <- var_77$sthrsw1[match(n77_symw %v% 'vertex.names',var_77$AID)]

n77_symw %v% 'slpenoughw1' <- var_77$slpenoughw1[match(n77_symw %v% 'vertex.names',var_77$AID)]

n77_symw %v% 'r_pubertyw1' <- var_77$r_pubertyw1[match(n77_symw %v% 'vertex.names',var_77$AID)]

n77_symw %v% 'pubertyw1raw' <- var_77$pubertyw1raw[match(n77_symw %v% 'vertex.names',var_77$AID)]

n77_symw %v% 'menarchew1' <- var_77$menarchew1[match(n77_symw %v% 'vertex.names',var_77$AID)]

n77_symw %v% 'gradew1' <- var_77$gradew1[match(n77_symw %v% 'vertex.names',var_77$AID)]

n77_symw %v% 'agew1' <- var_77$agew1[match(n77_symw %v% 'vertex.names',var_77$AID)]

n77_symw %v% 'malew1' <- var_77$malew1[match(n77_symw %v% 'vertex.names',var_77$AID)]

n77_symw %v% 'peducw1' <- var_77$peducw1[match(n77_symw %v% 'vertex.names',var_77$AID)]

n77_symw %v% 'racew1' <- var_77$racew1[match(n77_symw %v% 'vertex.names',var_77$AID)]

n77_symw %v% 'racew1_alt' <- var_77$racew1_alt[match(n77_symw %v% 'vertex.names',var_77$AID)]

n77_symw %v% 'white' <- var_77$white[match(n77_symw %v% 'vertex.names',var_77$AID)]

n77_symw %v% 'black' <- var_77$black[match(n77_symw %v% 'vertex.names',var_77$AID)]

n77_symw %v% 'natam' <- var_77$natam[match(n77_symw %v% 'vertex.names',var_77$AID)]

n77_symw %v% 'asianpi' <- var_77$asianpi[match(n77_symw %v% 'vertex.names',var_77$AID)]

n77_symw %v% 'othernat' <- var_77$othernat[match(n77_symw %v% 'vertex.names',var_77$AID)]

n77_symw %v% 'hisp' <- var_77$hispanicw1[match(n77_symw %v% 'vertex.names',var_77$AID)]

n77_symw %v% 'zbmiowobw1' <- var_77$zbmiowobw1[match(n77_symw %v% 'vertex.names',var_77$AID)]

n77_symw %v% 'fr_flag' <- var_77$fr_flag[match(n77_symw %v% 'vertex.names',var_77$AID)]

n77_symw %v% 'degree' <- degree(n77_symw, cmode = "freeman")

#recode physical development to 5-category

var_77$physdev5[var_77$pubertyw1raw < 2 ] <- 1 # 1-1.999

var_77$physdev5[var_77$pubertyw1raw >= 2 & var_77$pubertyw1raw < 3 ] <- 2 # 2-2.9999

var_77$physdev5[var_77$pubertyw1raw >= 3 & var_77$pubertyw1raw < 4 ] <- 3 # 3-3.9999

var_77$physdev5[var_77$pubertyw1raw >= 4 & var_77$pubertyw1raw < 5 ] <- 4 # 4-4.9999

var_77$physdev5[var_77$pubertyw1raw == 5 ] <- 5 # 5

var_77$physdev5<-as.numeric(var_77$physdev5)

# Attach new attributes to the network

n77_symw %v% 'physdev5' <- var_77$physdev5

#identify 9th-graders who remain from merged dyad/covar file:

gradeids<-as.data.frame(get.vertex.attribute(n77_symw,'gradew1'))

#Remove vertices, leaves us with n=1411 vertices w/full covar data

delete.vertices(n77_symw, c(71,999,1247,1259))

**4. Sample ERGM model**

#Full model (Table 2, column 1)

fullmodel <- ergm(n77_symw~ edges

+ gwdegree(0.2, fixed = TRUE)

+ gwesp(0.2, fixed = TRUE)

+ nodecov('physdev5') + nodecov('r_pubertyw1')

+ nodecov('pawkw1') + nodecov('slphrsw1') + nodefactor('slpenoughw1')

+ nodefactor('zbmiowobw1')

+ nodecov ('sthrsw1')

+ nodefactor('malew1')

+ nodecov('gradew1') + nodecov('agew1') + nodecov('peducw1') + nodecov('fr_flag')

+ nodefactor('black') + nodefactor('asianpi') + nodefactor('othernat')

+ nodefactor('hisp')

+ nodematch('malew1') + nodematch('gradew1', diff=T)

+ nodematch('zbmiowobw1')

+ nodematch('racew1_alt', diff=T) + nodematch('hisp')

+ absdiff('physdev5') + absdiff('r_pubertyw1')

+ absdiff('pawkw1') + absdiff('slphrsw1') + nodematch('slpenoughw1')

,

control=control.ergm(seed=5, MCMC.interval = 5000,

MCMC.samplesize = 2300, MCMLE.maxit = 30))

summary(fullmodel)

**5. ERGM diagnostics & plots**

#collinearity check (drawing from list of all models)

VIF.ERGM(fullmodel)

#GOF tests (drawing from list of all models)

gof_fullmodel<-gof(fullmodel] ~ degree + espartners + distance + model)

#print GOF plots

plot(gof_fullmodel)

#MCMC diagnostics

mcmc.diagnostics(gof_fullmodel)

**Part 2. Goodness of Fit for Sunshine High and Jefferson High models**

The examination of model goodness-of-fit in this paper is by using the suite of goodness-of-fit (GOF) functions provided in the ERGM package (degree, edgewise shared partners, geodesic distance, and Markov Chain Monte Carlo/MCMC diagnostic plots). To assess comparative fit between models, we compare the Akaike Information Criterion (AIC) between models [1] estimated in the [ergm] and [statnet] suite of packages [2]. Another goodness-of-fit indicator in this research is the variance inflation factor (VIF) from ERGM parameters [3]. Examination of VIF benefits the research by detecting collinearity-type problems and ensuring the consistency of model estimates.

**References**

1. Hunter DR, Handcock MS, Butts CT, Goodreau SM, Morris M. ergm: A package to fit, simulate and diagnose exponential-family models for networks. Journal of statistical software. 2008;24(3):nihpa54860. doi: 10.18637/jss.v024.i03. PubMed PMID: 19756229; PubMed Central PMCID: PMCPMC2743438

2. Handcock MS, Hunter DR, Butts CT, Goodreau SM, Morris M. statnet: Software tools for the representation, visualization, analysis and simulation of network data. Journal of statistical software. 2008;24(1):1548. doi: 10.18637/jss.v024.i01. PubMed PMID: 18618019; PubMed Central PMCID: PMCPMC2447931.

3. Duxbury SW. Diagnosing Multicollinearity in Exponential Random Graph Models. Sociol Method Res. 2021;50(2):491-530. doi: 10.1177/0049124118782543. PubMed PMID: WOS:000640002900002.

**S1 Table. VIF tests (Sunshine High & Jefferson High, main model in manuscript Table 2).**

| Table S1. Variance Inflation Factor (VIF) coefficients | | |  |
| --- | --- | --- | --- |
|  |  |  |  |
|  | Sunshine | Jefferson |  |
| *Endogenous network terms* |  |  |  |
| GW Degree (0.2) | 1.2 | 1.1 |  |
| GW Edgewise Shared Partners (0.2) | 1.2 | 1.1 |  |
| *Puberty and physical behaviors* |  |  |  |
| Puberty: Physical dev | 1.0 | 1.6 |  |
| Puberty: Relative timing | 1.2 | 1.3 |  |
| Physical activity, times per week | 1.3 | 1.2 |  |
| Sleep duration, avg. hours/night | 1.4 | 1.3 |  |
| Sufficient sleep | 1.4 | 1.8 |  |
| *Confounders* |  |  |  |
| Overweight/Obese | 1.6 | 2.6 |  |
| Screen time scale, hours per week | 1.1 | 1.1 |  |
| Male | 1.2 | 1.1 |  |
| Grade | 18.1 | 28.9 |  |
| Age | 4.8 | 10.0 |  |
| Parent highest education | 2.6 | 1.1 |  |
| Nomination error flag | 1.0 | 1.0 |  |
| Race: Black NH (Ref: White NH) | 25.0 | - |  |
| Race: AsAm/PI NH | 51.1 | - |  |
| Race: Other NH | 29.1 | - |  |
| Hispanic | 17.4 | - |  |
| *Dyadic homophily terms* |  |  |  |
| Same, Male | 1.0 | 1.0 |  |
| Same, White NH | 3.8 | - |  |
| Same, Black NH | 13.4 | - |  |
| Same, AsAm/PI NH | 21.0 | - |  |
| Same, Other NH | 7.5 | - |  |
| Same, Hispanic | 1.5 | - |  |
| Similarity, Pub (Phys Dev) | 1.0 | 1.1 |  |
| Similarity, Pub (Relative Timing) | 1.0 | 1.1 |  |
| Similarity, Physical activity | 1.1 | 1.0 |  |
| Similarity, Sleep duration | 1.0 | 1.1 |  |
| Same, Sufficient sleep | 1.1 | 1.7 |  |
| Same, Overweight/Obese | 1.5 | 2.5 |  |
| Same, 9th grade | - | 9.9 |  |
| Same, 10th grade | 7.1 | 2.4 |  |
| Same, 11th grade | 1.9 | 3.0 |  |
| Same, 12th grade | 6.7 | 7.2 |  |
| Note: Higher values indicate greater correlation.VIF > 20 is concerning, VIF > 100 indicates severe collinearity. | | |  |
|  |  |  |  |
|  |  |  |  |

**S1a Fig. GOF plots (Sunshine High, main model in manuscript Table 2).** Degree term is within bounds throughout the distribution; triadic term related to geometrically-weighted edgewise shared partners is suboptimal at values of 1-5, and geodesic distance is relatively ill-fit throughout the distribution.

**

**

**S1b Fig. MCMC plots (Sunshine High, main model in Table 2)***.* Density plots for all covariates are roughly normal and centered around zero.

**

**

**

**

**

**

**

**

**

**S2a Fig. GOF plots (Jefferson High, main model in manuscript Table 2).** Degree term is within bounds throughout the distribution; triadic term related to geometrically-weighted edgewise shared partners is suboptimal at values of 1-8, and geodesic distance is relatively ill-fit throughout the distribution above values of 2.

**S2b Fig. MCMC plots (Jefferson High, main model in manuscript Table 2).** Density plots for most covariates are roughly normal and most are centered below zero, indicating a poorer fit than at Sunshine High. Though this model is less well-fit, in the interest of having comparable covariates across models we retained the same terms.
