# Supplementary material for: Disentangling associations between pubertal development, healthy activity behaviors, and sex in adolescent social networks
Source: PLoS One. 2024 May 16;19(5):e0300715. doi: 10.1371/journal.pone.0300715 (PMC11098364; doi:10.1371/journal.pone.0300715)
Supplement: Attachment — Submitted filename: Response To Reviewers.pdf [file pone.0300715.s002.pdf]

Dr. Yasas Chandra Tanguturi
Editor: PLOS ONE

January 31, 2024

Dear Dr. Yasas Chandra Tanguturi,

We have completed revisions to our manuscript entitled "Disentangling associations between pubertal development, healthy activity behaviors, and sex in adolescent social networks" (PONE-D-23-37858) and we are pleased that a resubmission was invited for possible publication in *PLOS ONE*. We want to thank the editor and five reviewers for their close reads and constructive and thoughtful comments on our manuscript, and we think the manuscript is stronger as a result of considering their questions. Below we explain how editor (Part 1) and reviewers' (Part 2) comments have been addressed in this version of the manuscript, with our responses in bold.

Sincerely,
Mark Pachucki
o/b/o authors

****

Part 1 (Editor comments)
1. When submitting your revision, we need you to address these additional requirements. Please ensure that your manuscript meets PLOS ONE's style requirements, including those for file naming. The PLOS ONE style templates can be found at
https://journals.plos.org/plosone/s/file?id=wjVg/PLOSOne_formatting_sample_main_body.pdf and
https://journals.plos.org/plosone/s/file?id=ba62/PLOSOne_formatting_sample_title_authors_affiliations.pdf.
Authors: Thank you for this guidance. We have updated both the manuscript style format, as well as the title/authors/affiliations format to comply.
• In the manuscript, this mostly consisted of modifying headers, using proper bibliographic styles, correct equation numbering, and line numbering.
• In the title/authors/affiliations page, this consisted of adding a "¶" symbol to designate co-equal contributions of several authors.

2. Thank you for stating the following financial disclosure:
[Research reported in this study was supported by the National Institute of Nursing Research under Award Number R21NR017154 to Co-PIs Mark C. Pachucki and Lindsay T. Hoyt.]. Please state what role the funders took in the study. If the funders had no role, please state: ""The funders had no role in study design, data collection and analysis, decision to publish, or preparation of the manuscript."" If this statement is not correct you must amend it as needed. Please include this amended Role of Funder statement in your cover letter; we will change the online submission form on your behalf.
Authors: Thank you for this question; we have amended our funding statement, and included in our cover letter as well to say the following (below). Note that our manuscript was supported by a single NIH grant mechanism (R21NR017154). However, the data collection for the parent study (going back to 1994) was supported by three other federal grant mechanisms. As a condition of data use by the data provider, any author that uses these data is required to acknowledge the funding mechanisms for the parent study as well.

Research reported in this study was solely supported by the National Institute of Nursing Research under Award Number R21NR017154 to Co-PIs Mark C. Pachucki and Lindsay T. Hoyt. As a condition of data use, we acknowledge that this research uses data from the National Longitudinal Study of Adolescent to Adult Health (Add Health). Add Health is directed by Robert A. Hummer and funded by the National Institute on Aging cooperative agreements U01AG071448 (Hummer) and U01AG071450 (Allison E. Aiello and Hummer) at the University of North Carolina at Chapel Hill. Waves I-V data are from the Add Health Program Project, grant P01HD319121 (Harris) was directed by Kathleen Mullan Harris and designed by J. Richard Udry, Peter S. Bearman, and Kathleen Mullan Harris at the University of North Carolina at Chapel Hill, and funded by Grant P01HD31921 from the Eunice Kennedy Shriver National Institute of Child Health and Human Development, with cooperative funding from 23 other federal agencies and foundations. No direct support was received from grant P01HD31921, U01AG071450, or U01AG071448 for this analysis. The funders had no role in the current manuscript's study design, analysis, decision to publish, or preparation of the manuscript.

3. We note that you have indicated that there are restrictions to data sharing for this study. PLOS only allows data to be available upon request if there are legal or ethical restrictions on sharing data publicly. For more information on unacceptable data access restrictions, please see http://journals.plos.org/plosone/s/data-availability#loc-unacceptable-data-access-restrictions. Before we proceed with your manuscript, please address the following prompts:
a) If there are ethical or legal restrictions on sharing a de-identified data set, please explain them in detail (e.g., data contain potentially identifying or sensitive patient information, data are owned by a third-party organization, etc.) and who has imposed them (e.g., a Research Ethics Committee or Institutional Review Board, etc.). Please also provide contact information for a data access committee, ethics committee, or other institutional body to which data requests may be sent.
Authors: We have expanded our "data availability" statement to read as follows:

> **This research uses third party data from the National Longitudinal Study of Adolescent to Adult Health (Add Health). Information on how to obtain the Add Health data files is available on the Add Health website (http://www.cpc.unc.edu/addhealth). To apply for these data, the researcher must request access and download a contract application from the UNC Carolina Population Center Data Portal (https://data.cpc.unc.edu/projects/2/view). Once completed, researchers must submit their application through the portal. Data used in the current study were not collected by the study authors and therefore we do not have the permission to share the data. The data used are restricted use data from the Add Health study. Researchers who wish to gain access to this data must apply for access using the information specified above. The authors did not have any special access privileges to the data and confirm that the data was obtained in the same manner outlined above. Relevant codes for replicating analyses are within the Supporting Information files of this manuscript.**

In crafting this availability statement, we followed guidance of recent scholars who have recently published in PLOS ONE using these restricted data as well (Knox & TenEyck 2023; Jose et al. 2021; Verweij & Keizer 2022), similar to open-access policies in other publications such as Nature, Nature Human Behaviour, Nature Communications, PNAS, and Scientific Reports (we can provide a more comprehensive citation list if editors require it).

References:
Knox, Krysta N. & TenEyck, Michael F. (2023). Beauty is only skin deep: An examination of physical attractiveness, attractive personality, and personal grooming on criminal justice outcomes. *PLOS ONE.* vol. 18 (10), PMCID: PMC10566695
Jose, R.; Hipp, J. R.; Butts, C. T.; Wang, C.; & Lakon, C. M. (2021). A multi-contextual examination of non-school friendships and their impact on adolescent deviance and alcohol use. *PLoS One.* vol. 16 (2) , PMCID: PMC7875427
Verweij, R. M. & Keizer, R. (2022). The intergenerational transmission of educational attainment: A closer look at the (interrelated) roles of paternal involvement and genetic inheritance. *PLoS ONE.* vol. 17 (12 December) , PMCID: PMC9744317

4. Please include captions for your Supporting Information files at the end of your manuscript, and update any in-text citations to match accordingly. Please see our Supporting Information guidelines for more information: http://journals.plos.org/plosone/s/supporting-information.
Authors: Thank you. We have now appended a new subhead and caption for our Supporting Information file at the end of the manuscript (page 28, lines 2-4).

5. Please review your reference list to ensure that it is complete and correct. If you have cited papers that have been retracted, please include the rationale for doing so in the manuscript text, or remove these references and replace them with relevant current references. Any changes to the reference list should be mentioned in the rebuttal letter that accompanies your revised manuscript. If you need to cite a retracted article, indicate the article's retracted status in the References list and also include a citation and full reference for the retraction notice.

Authors: We have reviewed the reference list for completeness and accuracy, and we have confirmed that there are no retracted papers cited.

****

Part 2 (Reviewer comments)

We thank the reviewers for their helpful suggestions. In what follows, we respond point-by-point to reviewer concerns, with text additions underlined and italicized. We add numbers to each critique if reviewers did not include numbers, indicated with [#].

Reviewer #1: This article presents a scientific approach to identifying key factors establishing friendships in pubertal age group adolescents. Network analysis posits an interesting approach to analyze this sociological phenomenon. The authors summarize a vast evidence base of literature on physical activity and sleep which lays good groundwork for the study. My review points are:

1. In the first line of the introduction, the authors may want to change the word "outsize".
Authors: We appreciate R1's introductory comments praising our approach (network analysis) and synthetic overview of relational health behavior research on physical activity and sleep. We have changed the word "outsize" to "disproportionate".

2. Could the authors please explain how pubertal timing affected sleep activity in the Hoyt et al study, 2020.
Authors: Thank you for this question. We have now updated p7 to include more detail about how pubertal timing affected sleep activity in Hoyt et al. (2020). The revised text now reads, *"However, there was also some evidence that later timing is associated with health promoting (e.g., more sleep) activity behaviors [41]. Specifically, for both boys and girls, later adolescent physical development was prospectively associated with reporting more sleep nightly as young adults."*

3. The authors may want to expand on network analysis basics for the naive reader.
Authors: This is a welcome suggestion; indeed, on reflection, our analytic approach section launched into a highly technical form of social network analysis before giving a more general introduction. With space limitations in mind, we direct readers to useful guides and have revised this section (p15) to now read: *"Social network analysis (SNA) is a field of inquiry that seeks to explain the causes and consequences of patterned social relationships among humans; a wealth of useful guides are readily accessible [e.g. 62, 63, 64]. A key feature of SNA is a practical and statistical concern not just for individual attributes and qualities, but also a concern with quantities and qualities of ties between individuals and how – together – individuals and their social ties are connected with a given phenomenon. One type of SNA, exponential random graph models (ERGMs), were used to test study hypotheses and estimate determinants..."*

4. The study draws on data from the 1994-1995 study, a timeline when millenials were in the puberty. Given that we are in a different generation now, the authors may want to add this as a limitation for the generalizability of the study. Also, the use of devices have changed over time. Authors may wat to comment on this.
Authors: We appreciate the reviewer's concern about generational differences between now and youth at the time of data collection, and we have further expanded upon our statements about these generational differences (p14) to have a revised subheader: *"Considerations regarding modern adolescent socialization, pubertal development, and activities."* In this section we now note: *"In terms of pubertal development, the onset of puberty has rapidly declined since the industrial revolution [59] and has continued to decline (albeit more slowly) during the past several decades [60, 61]."*
• We agree that the use of devices have changed over time, and we expand upon our prior statement (p14) that, *"It is important to note that how adolescents socialize in today's smartphone era differs considerably from the 1990s…"* and append the statement that *"Screens are now ubiquitous in a way that is qualitatively different from youth experiences with visual media in the 1990s."*

References

59. Patton GC, Viner R. Pubertal transitions in health. The Lancet. 2007;369(9567):1130-9.
60. Eckert-Lind C, Busch AS, Petersen JH, Biro FM, Butler G, Bräuner EV, et al. Worldwide Secular Trends in Age at Pubertal Onset Assessed by Breast Development Among Girls A Systematic Review and Meta-analysis. Jama Pediatrics. 2020;174(4). doi: ARTN e195881
10.1001/jamapediatrics.2019.5881. PubMed PMID: WOS:000526597100002.
61. Parent A-S, Teilmann G, Juul A, Skakkebaek NE, Toppari J, Bourguignon J-P. The timing of normal puberty and the age limits of sexual precocity: variations around the world, secular trends, and changes after migration. Endocrine Reviews. 2003;24(5):668-93.

5. The assessment of pubertal development seems to be limited due to the tool used and the authors may want to allude to this as well.
Authors: We have expanded upon our prior statement in the limitations section of the discussion to be more explicit about this point. The revised text now reads,*"Although we are limited in what we can infer from the self-reported measures of puberty (e.g. Add Health does not include physician-administered Tanner staging or hormone samples) and we have no information on pubertal tempo (which requires repeated puberty assessment starting in childhood), the pubertal development measures have adequate variation, and these measures have been examined in high-quality analyses exploring pubertal development [20, 27, 54]"*

6. The discussion may further expand on the utility of these findings in a greater way.
Authors: Thank you for this comment – in conjunction with comments by R2, R3, and R4, we have significantly expanded on the conclusions to draw the links to practical take-aways for readers and to contextualize why findings from a study conducted with 1994-95-era youth is still relevant today.

7. The authors may want to add a limitations section to ennumerate the limitations related to the date of the data collection and possibly add what steps may be taken to possibly replicate the study in todays pubertal adolescents in order to have some more impactful results that have further value.
Authors: We agree with the reviewer that being specific about limitations of the data due to its date of collection are important; this was a critical observation from R3 as well.
• We have substantially expanded upon our thinking about secular changes in screen time usage and physical behaviors, as well as changes in puberty timing since the 1990s may affect what we can infer (p24, p26-27)
• On p24 we had stated, "Participants' self-report behavior measures also reflect the era in which they were gathered (1994-95), prior to both the widespread dissemination of devices to measure PA and sleep, and prior to the emergence of portable devices with screens. While activity type and frequency are less precise than current standards, these measures have been widely scrutinized and are similar to those in U.S. surveillance data, such as NHANES."
• On p25 we state that "Future network research on puberty would benefit from more careful attention to pubertal timing and tempo, as well as how individuals of different sexes, genders, and ethnicities *think* about pubertal development differently." To this latter point, we have now appended a statement relevant to today's youth as follows: *"For instance, current studies that provide far more refined measurement of pubertal development and related neurobiological processes [84-86] could be augmented with high-quality dyadic friendship data [e.g., 87,88,89] to provide an opportunity to build upon what we have done here."*

If these recommendations are addressed, this paper may be suitable for publication but otherwise the paper possibly only has limited historical value and hence should be rejected.
Authors: We appreciate the close read and the 7 recommendations that Reviewer 1 has offered. To make our philosophical position clearer for readers, we have expanded upon our commentary regarding modern youth socialization (p14) to now explicitly state that, *"…we take the position that these analyses can be informative to the current era and not simply historical analysis, a point we return to in our conclusions."* In the conclusions section (p26-27) we expand upon this point considerably.

Reviewer #2: The paper attempts to explore an interesting area of research regarding the interactions between pubertal development, healthy activity behaviors, and social networks among adolescents. A few points to improve the article.

[1] The paper's extract lacks comprehensiveness by omitting the study's findings and conclusions. It focuses primarily on the abstract, introduction, and methods, which leaves a reader without a full understanding of the research's outcomes. There should be a different heading of Conclusions
Authors: Thank you for this comment. We will admit to being slightly confused by Reviewer 2's phrase "The paper's extract lacks comprehensiveness…" and would appreciate further guidance. If the reviewer meant "abstract", we have re-examined the abstract and do think that it offers a capsule summary of statement of our scientific problem, how and why we go about studying this problem, a brief statement of findings, and brief comment about implications. If this comment is related to the discussion, we also want to clarify that we have now added a new heading for *"Conclusions and Practical Applications"*" (related to R2, comment 4) to further expand on the study's findings and conclusions*"*

[2] Discuss the limitations of cross-sectional data in establishing cause-effect relationships. It is mentioned in one line only.
Authors: We appreciate that there are a variety of ways that cross-sectional data can limit our understanding of cause-effect relationships. We have revised our former statement (p24) as follows: *"A perennial observational research concern is reciprocal causation (i.e. whether friendship changes cause behavioral shifts, or whether behaviors lead to friendship). Without more granular longitudinal information on behavior and friendship changes to establish temporal ordering, a cross-section of yearly data can provide insight into associations, but cannot provide causally informative evidence of statistical relationships."*

[3] The absence of a discussion on the generalizability of findings diminishes the argument's broader relevance, especially when data is drawn from specific settings. Comment on this in the limitation as well.
Authors: We appreciate this concern, and we agree. We take the position that it is a strength to treat each school as having its own 'network ecology', following the work of McFarland and colleagues – and we do not make claims to generalizability to all schools. We have now appended a statement on p25 to, *"Yet it would be inappropriate to claim that our findings generalize to all schools. Given differences in school types, sizes, student composition, and cultures, care and thoughtfulness are warranted in applying these findings to school settings beyond the two types studied here. As McFarland and colleagues [43] point out in relational analyses of multiple school environments, giving attention to specific network ecologies of different schools is critical in considering how interventions might be implemented most successfully. Our study follows suit, demonstrating that it is important for the study of pubertal development to account for the network context of other physical health-promoting behaviors (PA, sleep) and risk factors (ST, weight status) that may independently shape, as well as confound, puberty's relationship with friendship formation."*

[4] Discuss the practical implications and real-world applications as a separate heading to enhance its relevance beyond academic circles.
Authors: Thank you for this comment; we have endeavored to address this concern in tandem with the first comment.

Reviewer #3: Thank you for the opportunity to review the manuscript, "Disentangling associations between pubertal development, healthy activity behaviors, and sex in adolescent social networks." The study is a detailed analysis of how pubertal timing/development, physical health, sleep, and biological sex influence friendship formation in adolescents. The paper is very well-written and provides sufficient detail on the study, methods, and findings. Overall, this is an interesting paper that contributes to the literature by using a novel statistical approach to the question of whether puberty, physical health, and more influence friendship formation. With these strengths in mind, a few concerns remain.

[1] - Of most substantial concern is the timeline of the data collected. Specifically, as the authors highlight, the collected data is from 1994-1995. While the results and discussion presented are interesting, it is difficult to determine whether similar results would be found in today's adolescent population. With the social landscape of 1994-1995 being quite different from today (e.g., prevalence of social media, online friendships, etc.), it would helpful for the authors to discuss how their findings may be translated to be relevant for adolescents in 2023. Similarly, the average age of pubertal onset is younger today than 20-30 years ago- how might an even earlier pubertal onset for some further influence friendship formation and social connections?

Authors: We thank R3 for their concerns about the time at which the data were collected, a concern shared by R1, and by our team as well. Carefully reconsidering the possibilities has led to insights that we think enhance the paper appreciably. It is indeed an interesting question whether similar results would be found among today's youth, and ultimately, we argue that (a) our associational findings are likely to be conservative relative to today's youth, and (b) we hope that one contribution of this study is to provide a roadmap for one way to study the associations (and hopefully, prospective associations in a longitudinal framework) between physical development and friendships (p25-26). It bears noting that interpersonal mechanisms like social comparison, social desirability, and popularity existed then, and still drive a great deal of adolescent (to say nothing of adult!) behavior. We look forward to our colleagues in the field continuing to build datasets that would allow us to confirm this assumption; we now point out useful possibilities of extending existing data sources, and offer strong advocacy for this type of research with new text on p25 (line 23)-p26 (lines 1-3).

To our minds, there are intersecting issues here: changes in screen use, and changes in puberty timing which may have independent and interactive effects on friendship formation and maintenance. By themselves, each issue provokes several competing hypotheses. Taken together, it is difficult to predict specific outcomes, though we use the below thinking outlined below to more clearly point to this uncertainty and highlight some possibilities on p24-25 to help readers bridge between findings from data gathered several decades ago with the current moment. Though this kind of speculation is useful to provoke new hypotheses to test as extensions of this research, it is currently beyond the scope of this already somewhat lengthy paper to include the below hypothesizing in its entirety.

• (a) Secular changes in screen use: It is certainly plausible that today's youth are more densely connected to one another due to ubiquitous screens and online presence, though some might argue that at least some of this social connection is relatively insubstantial. Social network scholars have recently called for more attention to theorizing the causes and consequences of offline versus online friendships [56], a point we now reinforce on page 24.

(1) H1: One plausible hypothesis related to physical behaviors (PA, Sleep) is that that a greater extent of connection would amplify the magnitude of associations we see in terms of similarity of friends having similar physical behaviors.

(2) H2: A competing hypothesis might be that perhaps social connection is in some ways "thinner" due to, arguably, less (and perhaps less meaningful) in-person interactions, making youth less likely to gravitate to those with similar behaviors, leading to a lower extent of friendship.

• (b) Secular changes in puberty timing: As to the declining age of puberty, we have now added an explicit statement first acknowledging this point on p14, "*<u>In terms of pubertal development, the onset of puberty rapidly declined since the industrial revolution [59] and has continued to decline, albeit more slowly, during the past several decades [60,61].</u>"* In terms of theorizing how earlier pubertal onset might influence friendship formation and social connections, we might hypothesize several scenarios.

(1) H1: uniform decline in pubertal timing does not lead to appreciable change in puberty similarity among friends due to uniform decline in pubertal timing: in one scenario, due to consistent declining onset across youth, similarity in pubertal timing among connected friends does not change meaningfully.

(2) H2: uniform decline in pubertal timing leads to more friendship turnover. Here we take inspiration from scholars of adolescent development [59, 80], who point out that looking at secular changes in pubertal development are not likely to be as productive as investigating the growing gap between one's age of

physical maturity (going down) and age of psychosocial maturity (going up). This widening gap might lead to an increase in risk-taking health behaviors and possibly riskier friendships made without thought or care, leading to more turnover, fewer solid connections, fewer "best friendships" and so forth. In some ways, the socialization loss currently being observed among youth who spent some part of 2020-2021 with impaired learning due to COVID-19 might give us leverage on the question of what might happen if ability to form social connections was inhibited/disrupted. Interestingly, we can see this uncertainty leading to less similarity on pubertal timing among friends, but inconsistency and uncertainty in socialization might prompt a kind of social development recession wherein youth would seek friends with some type of trait similarity, pubertal timing status included.

(3) H3: uniform decline in pubertal timing leads to less friendship turnover/more stability. Although it is possible, this scenario seems unlikely due to the widening gap between physical and psychosocial maturity mentioned in H2.

(4) H4: Non-uniform decline in pubertal timing leads to differential effects for subpopulations. If changes in onset of puberty and pubertal tempo are happening at different rates for girls and boys, and/or racial/ethnic groups, for instance, we might also hypothesize differential effects for subpopulations.

References

56. Lewis K. Digital networks: Elements of a theoretical framework. Soc Networks. 2021. doi: 10.1016/j.socnet.2021.12.002.

59. Patton GC, Viner R. Pubertal transitions in health. The Lancet. 2007;369(9567):1130-9.

60. Eckert-Lind C, Busch AS, Petersen JH, Biro FM, Butler G, Bräuner EV, et al. Worldwide Secular Trends in Age at Pubertal Onset Assessed by Breast Development Among Girls A Systematic Review and Meta-analysis. Jama Pediatrics. 2020;174(4). doi: ARTN e195881
10.1001/jamapediatrics.2019.5881. PubMed PMID: WOS:000526597100002.

61. Parent A-S, Teilmann G, Juul A, Skakkebaek NE, Toppari J, Bourguignon J-P. The timing of normal puberty and the age limits of sexual precocity: variations around the world, secular trends, and changes after migration. Endocrine Reviews. 2003;24(5):668-93.

80. Dahl RE. Adolescent brain development: a period of vulnerabilities and opportunities. Keynote address. Ann N Y Acad Sci. 2004;1021:1-22. Epub 2004/07/15. doi: 10.1196/annals.1308.001. PubMed PMID: 15251869.

[2] - The hypotheses are unclear. The entire "Hypotheses" section is quite long, and therefore, it would likely aid in reader comprehension to have a short, concise summary of the hypotheses at the end of the section to clearly define Hypothesis 1, 2, etc.

Authors: Thank you for this comment; we have now included a capsule restatement of hypotheses to aid readers on p10.

[3] - Are the authors able to present a means of interpreting the size of an effect in the Results for readers unfamiliar with the statistical approach. Especially as there are instances when the authors cite a "marginal" effect for a p-value of 0.08 (which some would argue is not marginal/trend). As such, providing a means to easily understand the effect size, rather than relying on p-values, would be beneficial.

Authors: We appreciate this concern, and we have tried to make the interpretation of coefficients more straightforward for readers unfamiliar with ERGM estimates with the addition of the following prose on p19:

> ***Because interpreting unadjusted ERGM coefficients is not necessarily straightforward for readers unfamiliar with this statistical approach, we contextualize that*** **the magnitude of these dyadic coefficients is relatively small. For instance, exponentiating the coefficient for similar relative pubertal timing among students at Sunshine High is equivalent to a 1.04 times greater likelihood of those individuals being friends, and similar sleep duration at Jefferson equivalent to a 1.03 times greater likelihood of friendship, compared with the 1.5 times greater likelihood of friendship among two students of the same sex.**

[4] - In the Discussion, the authors conclude the manuscript discussing what to do with this information. It seems more time could be spent focusing on this in the Discussion, as currently, one might come away from reading the manuscript still questioning the impact and relevance of these findings. For this reviewer personally, while I walked away from the manuscript thinking the results were intriguing, I was still left with wondering 'now what?' or 'so what?'.

Authors: Yes, we agree with this point. We hope that we now give a more coherent answer to a "so what" question with increased attention to the productive thinking illuminated by your first point above, and the suggestion of R2 which prompted us to distinguish a separate "Conclusion and Practical Application" section at the end.

Reviewer #4: This is a well-written manuscript. I liked reading this manuscript and believe that it is very promising. At the same time, I identified couple of issues that require the authors' attention. Introduction is too long and same goes with the conclusion and discussion which can be combined and shortened to improve the readability of the article. Professional use of English language is at par. Overall, this will be a good addition to current available literature.

Authors: We thank R4 for their encouragement that this study adds a well-written contribution to what is currently known. We would prefer brevity as well, and have reviewed the entire manuscript with a special focus on the introductory section(s) leading up to the hypotheses prompted by prior research, and have shortened by 200 words (from 2515 words to 2315 words). While we are also sympathetic to R4's request that the discussion/conclusion be consolidated because of length, the concerns brought forward by R1, R2, and R3 have led us to slightly extend the discussion (and more clearly separate the conclusions to account for a concise 'what now?' statement). We hope that these tradeoffs make the manuscript even more clear for readers.

Reviewer #5: Very interesting topic and study. It seems to be a topic that is difficult to study and have clear findings about given the number of confounders that are possible (e.g how close to they live, same route they take, where they sit in class, etc). Although there were not many associations found, there were a few that seem significant.

[1] -What do you account for the increased likelihood of friendships in young with similar sleep duration?
Authors: Thank you for this question, which made us realize we did not include a clear statement relaying our perspective. We now incorporate it on p21 as follows:

> **But we also replicated regression modeling-based trends towards youth with similar sleep duration [15] and, to a lesser extent, sleep sufficiency [40] selecting one another as friends *<u>with a more precise modeling technique that accounts for interpersonal statistical dependence. This makes sense intuitively, both in cases where friends are socializing in-person at night in ways that lead to a common shared bedtime, as well as cases where they are not together, given bedtime norms and the likelihood of youth engaging in similar evening behaviors, such as watching similar TV shows or talking on the phone</u>*. Taken together, these findings reinforce that a network perspective in sleep research continues to show promise.**

[2] -I don't see what were the age/grade ranges of the adolescents included in the ADD health study since you later mention perhaps older boys might show more association? Also, I'm sure grade level has a very strong association among friends. It might help to correct for grade? I'm not sure if that was done.
Authors: Thank you for this opportunity to clarify; yes, all estimates reported in Tables 2,3,4 adjust for both age and grade at the individual level, and within-grade friendships at the peer level. The age range of youth at School 1 (Sunshine High) was 15-21, μ 17.3 (SD 1.0), and School 2 (Jefferson High) was 15-20, μ 16.9 (SD 1.2) as reported in Table 1. To make it more apparent, we have amended the following on p17:

> **However, with regard to both pubertal development measures, participants at Jefferson High report being slightly more developmentally mature than those at Sunshine (both in terms of physical development, as well as perceptions of pubertal development relative to same-sex peers), which is curious given the younger average age of Jefferson participants *<u>(μ=16.9 and SD=1.2, relative to μ=17.3 and SD=1.0 at Sunshine)</u>*.**

[3] - How do you account for the differences between your findings and that of the Dutch study?
Authors: It is an interesting question, though we feel that speculation about the similarities or differences extends somewhat beyond the scope of this study. We had gone into analyses (p5) with the following speculation: "Speculatively, given sociocultural norms between the Netherlands (more Western social context) and Taiwan (more collectivistic), we might expect a closer fit with findings from the Dutch youth study." With this said, we have added some qualification to this point on p23:

> **Our work also comports more closely with the statistical network research of Franken and colleagues [19] who found that Dutch youth with similar pubertal development tended to be friends, rather than that of Lee and colleagues [24] who found no support for friendship based on pubertal development similarity among Taiwan youth. *<u>We would speculate that US-based youths' affinities with the Dutch case might owe more to youth socialization and friendship norms in Western societies rather than intercultural differences in pubertal timing, though more research is needed to assess this.</u>***
